# Mechanism of Coronary Microcirculation Obstruction after Acute Myocardial Infarction and Cardioprotective Strategies

**DOI:** 10.31083/j.rcm2510367

**Published:** 2024-10-12

**Authors:** Yuyu Li, Jiaqi Yu, Yuan Wang

**Affiliations:** ^1^Beijing Anzhen Hospital Affiliated to Capital Medical University, 100029 Beijing, China; ^2^Beijing Institute of Heart, Lung and Blood Vessel Disease, 100029 Beijing, China

**Keywords:** acute myocardial infarction, coronary microvascular dysfunction and obstruction, mechanism, cardioprotection

## Abstract

ST-segment elevation myocardial infarction patients are best treated with emergency percutaneous coronary intervention (PCI), while coronary microvascular dysfunction and obstruction (CMVO) are indicated by the absence or slowing of antegrade epicardial flow on angiography, resulting in suboptimal myocardial perfusion despite the lack of mechanical vascular obstruction. CMVO occurs in up to half of patients who undergo PCI for the first time and is associated with poor outcomes. This review summarizes the complex mechanisms leading to CMVO and elaborates on the changes observed at the organism, tissue, organ, cellular, and molecular levels. It also describes the current diagnostic methods and comprehensive treatment methods for CMVO.

## 1. Introduction

ST-segment elevation myocardial infarction (STEMI), which is usually caused by acute thrombotic occlusion of the coronary arteries, is a leading cause of heart failure and death [1]. Timely percutaneous coronary intervention (PCI) or reperfusion therapy with thrombolytic drugs can effectively save the at-risk myocardial tissue and reduce the infarct size [2]. The time to the hospital from the onset of chest pain symptoms for recanalization is extremely important. Although the time from admission to recanalization in patients with acute myocardial infarction (AMI) undergoing primary PCI has decreased significantly over the past few years, patient hospitalization and mortality rates have hardly changed [3]. Therefore, additional strategies are needed to reduce hospital mortality in this population.

PCI can be used to open the large blood vessels blocked by AMI, allowing blood to flow again and the tissue to be reperfused. However, in cases of coronary microvascular dysfunction and obstruction (CMVO) [2, 4], the blood vessel cannot be directly recanalized through interventional means. Even when the infarct-related artery is rapidly recanalized, CMVO can still occur [5]. The presence of coronary microvascular dysfunction can elevate the risk of cardiovascular events, regardless of whether there is epicardial disease or not. Meanwhile, the occurrence of coronary microvascular occlusion can increase the risk of cardiovascular events, regardless of whether there is macrovascular occlusion in the heart [6, 7]. Patients with pre-existing microvascular dysfunction benefit less from timely reperfusion of the great cardiac vessels during reperfusion of occluded coronary arteries than patients without pre-existing microvascular dysfunction, emphasizing that maintenance of normal microvascular function before acute coronary occlusion is a key goal for prevention [8].

## 2. Pathological Mechanism of Coronary Microcirculation Obstruction

CMVO refers to hypoperfusion of the myocardial tissue due to changes in the anterior arterioles (100–500 µm in diameter) and tiny blood vessels (diameter <100 µm) after epicardial coronary artery recanalization.

### 2.1 Individual Susceptibility

#### 2.1.1 Genetic Susceptibility

Genetic and acquired susceptibility to microvascular injury may play an essential role in the regulation of the no-reflow phenomenon. The pathogenic component of CMVO is an individual susceptibility to microvascular dysfunction, which may be related to microcirculatory function, structure, and density [9]. Moreover, genetic factors may modulate adenosine-induced vasodilation. A previous study have shown that the 1976T.C polymorphism of the gene encoding the adenosine A2A receptor (ADORA2A) may be associated with a higher incidence of CMVO [10]. Yoshino *et al*. [11] conducted a study on sex-specific single-nucleotide polymorphisms and their relationship with coronary microvascular dysfunction. The results showed that variations in certain regions of vascular endothelial growth factor A (VEGFA) and cyclin-dependent kinase inhibitor 2B antisense RNA (CDKN2B-AS1) are linked to this dysfunction. Additionally, myosin heavy chain 15 (MYH15), VEGFA, and 5’-nucleotidase ecto (NT5E) have allelic variants that are specific to men and increase the risk of coronary microvascular dysfunction [11].

#### 2.1.2 Acquired Susceptibility

Although genetically determined exposure to microcirculatory damage is difficult to avoid in actual clinical diagnosis and treatment, acquired vulnerability can be moderated and treated [12]. Sara *et al*. [13] found that poor glycemic control was associated with coronary microvascular dysfunction in a cohort of women with diabetes mellitus who presented with chest pain and non-obstructive coronary artery disease clinically. Further research is therefore needed to identify additional risk prevention tools and therapies targeting microvascular dysfunction as a composite indicator of cardiovascular risk [13]. Iwakura *et al*. [14] investigated whether preadmission statin therapy affects the development of the no-reflow phenomenon after infarction in patients with hypercholesterolemia. The study showed that long-term pretreatment with statins can effectively maintain the microvascular integrity after AMI without relying on lowering lipids, thereby improving the recovery of cardiac function [14].

### 2.2 Tissue and Organ Level

#### 2.2.1 Coronary Microarterial Embolism

Spontaneous rupture of atherosclerotic lesions or rupture of iatrogenic injuries in large- or medium-sized coronary arteries leads to the release of plaque fragments, which together with superimposed thrombus-forming substances can lead to embolism formation in the coronary microcirculation [15]. Experimental observations have shown that myocardial blood flow is irreversibly reduced when micro-thrombosis blocks more than 50% of the coronary capillaries [16]. Depending on the size of the fragments, physical obstruction of the coronary microcirculation results in typical microinfarction, which subsequently induces an inflammatory response [17]. Thus, a small number of emboli during primary PCI in patients with STEMI may create a local reactive environment while not affecting myocardial perfusion, leading to the release of inflammatory and vasoactive substances, such as endothelin-1 [15].

#### 2.2.2 Capillary Destruction and Hemorrhage

In the process of myocardial infarction, damage to the structure of the coronary microcirculation (that is, capillary rupture and hemorrhage) is the most severe manifestation of damage to the microcirculation. Endothelial cells swell substantially at this time, and the blood vessel wall ruptures. After the blood vessels have been recanalized, erythrocytes enter the gap between the myocardial tissue, and intramyocardial hemorrhage (IMH) occurs [18, 19]. The presence of erythrocytes in the interstitial space can lead to iron deposition, which further induces an inflammatory response and exacerbates defective reperfusion injury [20].

#### 2.2.3 Microvascular Diastolic Dysfunction

During myocardial ischemia, the coronary circulation is not maximally dilated. Instead, it has sustained vasoconstrictive tension, which can be eliminated by drugs, thereby improving local myocardial blood flow and contraction [21]. Microvascular diastolic dysfunction is mainly divided into (i) endothelium-dependent contraction (EDC) and (ii) non-endothelium-dependent contraction (non-EDC). Endothelial cell dysfunction occurs due to various factors, such as myocardial ischemia, hypoxia, and reperfusion injury. One of the early signs of such endothelial cell dysfunction is that the concentration of calcium in the cytoplasm of endothelial cells increases, which stimulates the production of prostaglandins and causes microvascular contraction [22]. In addition, endothelin-1 and angiotensin secreted by endothelial cells can cause abnormal vasoconstriction. Reducing vasodilatory factors secreted by endothelial cells, such as nitric oxide, is an essential factor causing diastolic microvascular dysfunction. Non-EDC often depends on direct regulation of sympathetic nerves, which are overactivated during myocardial ischemia and early reperfusion. At this time, activation of α-adrenergic receptors can lead to increased coronary vasoconstriction [23, 24, 25]. Some endothelial cell-independent vasoactive substances have also been discovered in recent years. For example, Herring *et al*. [26] found that elevated coronary sinus neuropeptide Y concentrations are directly associated with increased microvascular resistance index, edema, and microvascular obstruction in patients with reperfusion after AMI.

In addition, ischemia–reperfusion injury can lead to endothelial cell dysfunction and promote endothelial cell apoptosis by affecting mitochondrial fusion, excessive division, and autophagy of vascular endothelial cells, thereby leading to CMVO and IMH [27]. Vascular endothelial dysfunction leads to the overexpression of adhesion molecules, such as vascular cell adhesion protein-1 and intercellular adhesion molecule-1. Vascular endothelial dysfunction also leads to the release of many inflammatory factors, such as tumor necrosis factor-α and interleukin-1β, as well as metabolic molecules, such as prostaglandins, endothelin-1, and angiotensin [28, 29, 30]. These factors aggravate ischemia–reperfusion injury in the heart and promote the occurrence of cardiac microcirculation disturbance.

### 2.3 Cellular and Molecular Level

#### 2.3.1 Cell Edema

Cardiomyocytes are often more sensitive to hypoxia than other cell types [31]. Cardiomyocyte edema caused by myocardial ischemia/reperfusion compresses the coronary microcirculation, aggravates damage to the microcirculation, and leads to hypoperfusion, culminating in a vicious cycle [32, 33]. These factors aggravate ischemia–reperfusion injury in the heart and promote the occurrence of cardiac microcirculation disturbance.

#### 2.3.2 Increased Neutrophil Adhesion and Release of Neutrophil Extracellular Traps

In the process of AMI, neutrophils, as the immune vanguard of the body, can reach the site of inflammation, with the neutrophil recruitment peak occurring 24 hours after myocardial infarction [34]. The peak of CMVO occurs 1–2 days after myocardial infarction [35]. Several clinical cohort studies have shown that the number of neutrophils after myocardial infarction positively correlates with the area (severity) of CMVO [34, 36]. El Amki *et al*. [37] showed that neutrophils directly block the microvasculature, and the Ly6G antibody effectively alleviates the occurrence of microcirculation disorders. In addition, increased neutrophil recruitment in the heart combines with the overexpression of adhesion molecules on activated microvascular endothelial cells. This process directly leads to blockage of the microcirculation, aggravating CMVO. Centrioles also promote the formation of micro-thrombosis in arterioles by releasing neutrophil extracellular traps (NETs), triggering the occurrence and development of the no-reflow phenomenon [38].

#### 2.3.3 Platelets and Their Metabolites

**Fig. 1.**
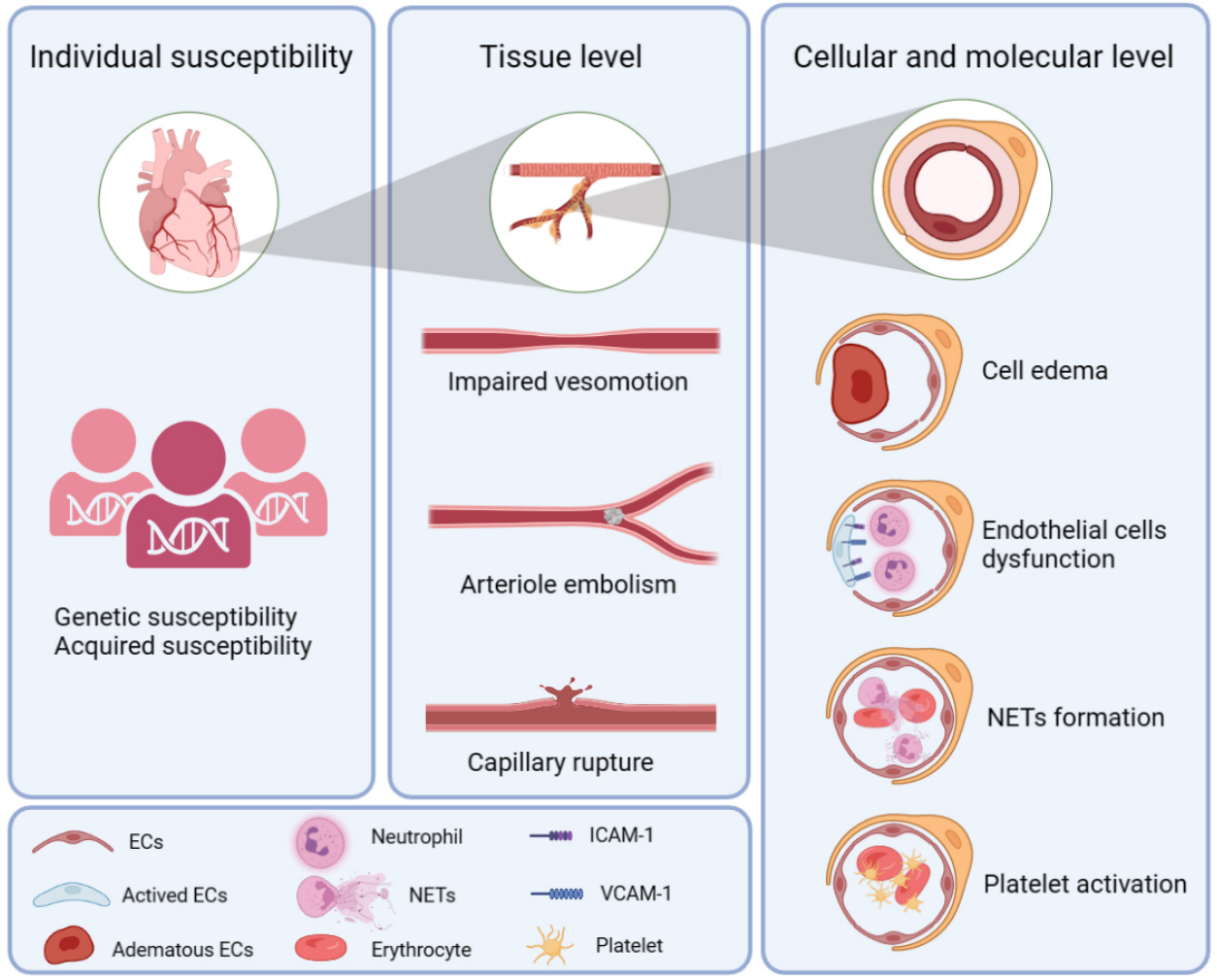
**Pathological mechanism of coronary microcirculation obstruction**. NETs, neutrophil extracellular traps; ECs, endothelial cells; ICAM-1, intercellular cell adhesion molecule-1; VCAM-1, vascular cell adhesion molecule-1.

Coronary microvascular constriction is caused by platelets and other substances that are released upon activation, including adenosine diphosphate, serotonin, and thromboxane A2 [39]. To some degree, protecting the coronary microcirculation can be achieved by inhibiting platelet activation. Following myocardial ischemia-reperfusion, the expression of adhesion molecules is elevated in both the coronary vascular system and circulating cells, which leads to platelet and leukocyte adhesion to the endothelium and the formation of platelet-leukocyte aggregates [40]. Adherent cells and aggregates affect coronary microvascular blood flow through physical blockade. Characteristic erythrocyte aggregates can obstruct capillary circulation when there is a reduction in coronary microvascular blood flow (Fig. 1) [41].

#### 2.3.4 Pericytes Contraction

Pericytes are tightly present around the septum of capillaries and play an important role in stabilizing the blood-brain barrier, regulating blood flow, and immunomodulation, and the continued contraction of pericytes ultimately leads to impaired blood flow and poor clinical outcomes in ischemic stroke [42]. In a number of studies on the microvascular function of the heart and brain, it is effective in improving cardiac microcirculation disorders and regulating stroke by inhibiting microvascular pericyte contraction [43, 44].

## 3. Diagnosis for CMVO

### 3.1 Invasive Examination

#### 3.1.1 Coronary TIMI Blood Flow Grading 

**Fig. 2.**
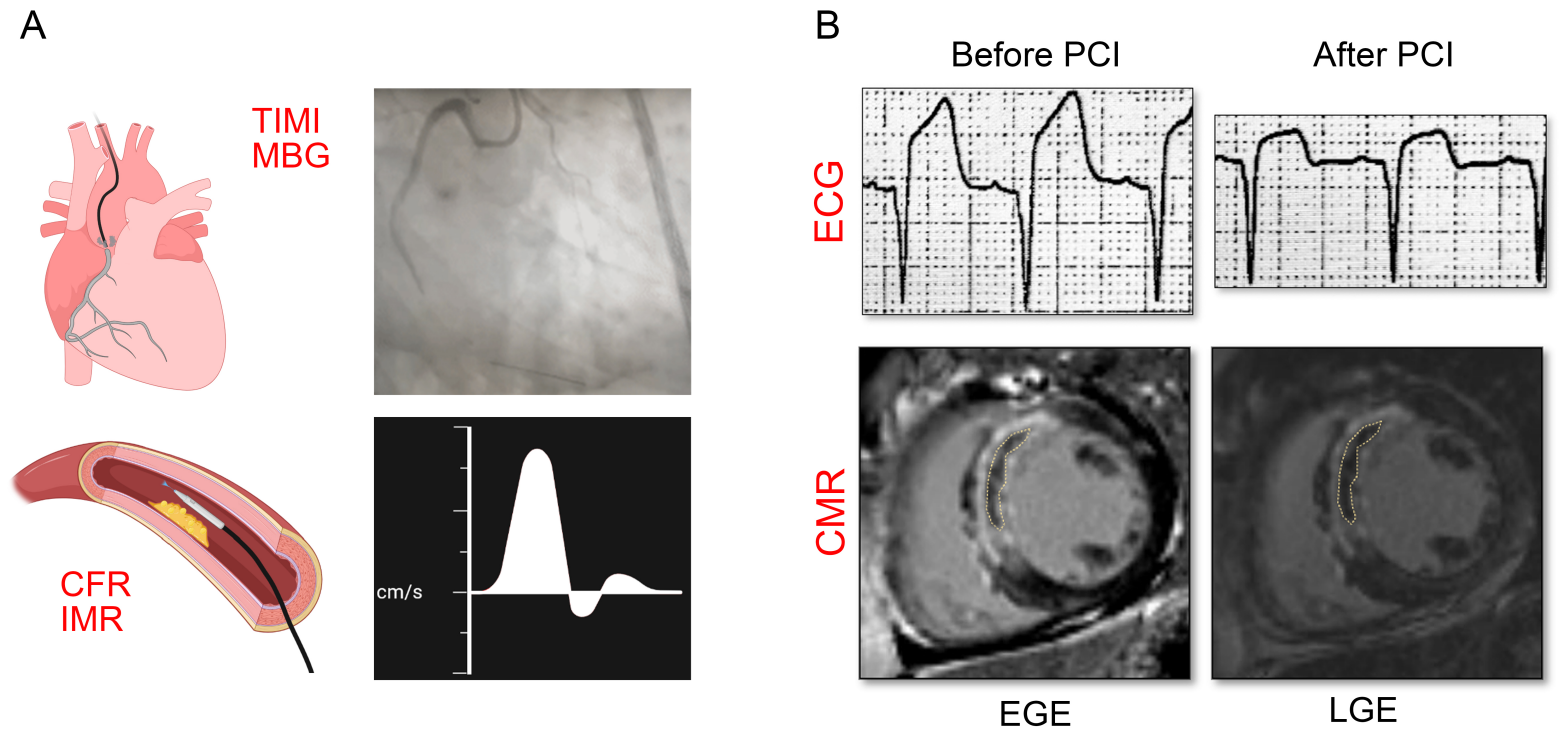
**Instrument examination of CMVO**. (A) Invasive examination of CMVO. TIMI flow grade, MBG, CFR, and IMR are the main measures used to diagnose CMVO. (B) Non-invasive examination of CMVO. Electrocardiography, a common non-invasive examination, can be used to evaluate CMVO by comparing ST-segment elevation before and after PCI. CMVO, coronary microvascular obstruction; TIMI, thrombolysis in myocardial infarction; MBG, myocardial blush grade; CFR, coronary flow reserve; IMR, index of microcirculatory resistance; PCI, percutaneous coronary intervention; ECG, electrocardiogram; CMR, cardiovascular magnetic resonance; EGE, early gadolinium enhancement; LGE, late gadolinium enhancement.

Sheehan *et al*. [45] reported for the first time in the thrombolysis in myocardial infarction (TIMI) trial that the visual score of coronary angiography ranges from 0 (no reperfusion) to 3 (complete reperfusion). The TIMI flow grade is the most commonly used assessment of coronary reperfusion. TIMI blood flow grade 0–1 suggests no reflow, grade 2 suggests slow blood flow, and grade 3 suggests normal blood flow. However, TIMI grade 3 only indicates normal epicardial flow, not normal myocardial perfusion [46]. The TIMI perfusion grade (TMPG) is an index that is used to judge myocardial perfusion according to the morphology and duration of contrast agent in the myocardial tissue. The TMPG is divided into grades 0–3. TMPG 3 suggests improved perfusion at the myocardial level, and the incidence of adverse events after primary PCI is lower than in patients with grade 0–2 STEMI [47] (Fig. 2A).

#### 3.1.2 Myocardial Blush Grade

The myocardial blush grade (MBG) is used to assess myocardial perfusion in the catheterization laboratory and is divided into three grades (0–3), with grade 0 indicating no myocardial color, grade 1 indicating a little myocardial color, grade 2 indicating moderate myocardial color, and grade 3 indicating normal myocardial color (the same color as the non-infarct-related artery blood supply area). MBG 0–1 suggests no myocardial perfusion, grade 2 suggests partial myocardial reperfusion, and grade 3 suggests complete myocardial reperfusion [48]. MBG 2–3 is associated with less microvascular occlusion than MBG 0–1, as well as a lower mortality rate 3 days after surgery in patients with STEMI [49] (Fig. 2A).

#### 3.1.3 Coronary Flow Reserve and the Index of Microcirculatory Resistance

The use of guidewires can realize the measurement of coronary flow reserve (CFR) and the index of microcirculatory resistance (IMR). A CFR of <2.0 in maximal myocardial hyperemia can be judged as impaired coronary microcirculation when epicardial vascular stenosis resolves without spasm [50]. IMR is a reliable indicator for quantitative analysis of coronary microcirculatory resistance. An IMR of <25 is generally considered normal, and an IMR of >40 is associated with a higher rate of clinical events. Approximately 33% of patients with STEMI who have a postoperative IMR of >40 after PCI have significantly increased rates of heart failure rehospitalization and mortality, as well as a worse long-term prognosis [51] (Fig. 2A).

#### 3.1.4 Quantitative Blood Flow Microvascular Resistance 

CFR and IMR are obtained by three-dimensional vascular reconstruction and hemodynamic analysis of coronary angiography without a pressure guidewire or induced hyperemia [52, 53]. The results of the early MYO-QFR study showed that a contrast flow rate QFR-fixed flow rate QFR (cQFR-fQFR) of >0.07 or a contrast flow rate of <0.1 in patients with STEMI may be associated with coronary microvascular dysfunction (CMD) [54]. Therefore, quantitative blood flow microvascular resistance (QFR-MR) is expected to become a new means to detect resistance in the microcirculation.

### 3.2 Non-Invasive Examination

#### 3.2.1 Electrocardiography

After the initial PCI, incomplete ST resolution (STR) is associated with CMVO and poor clinical outcomes [55, 56]. In a recent study, ST-segment elevation after PCI was an independent marker of CMVO, and STR was absent in approximately one-third of TIMI grade 3 and MBG 2–3 patients. According to a previous study, the simple measure of the maximal residual degree of ST-segment elevation (STE) after primary PCI is a strong independent predictor of both survival and freedom from reinfarction at 30 days and 1 year [57] (Fig. 2B).

#### 3.2.2 Cardiac Magnetic Resonance Imaging 

Cardiac magnetic resonance imaging (CMRI) is a non-invasive diagnostic tool that uses non-ionizing radiation and is widely used in patients with STEMI [58, 59]. Perfusion of the infarct core ceases due to obstruction, loss of vascular integrity, and bleeding, resulting in low enhancement of the infarct core in the necrotic zone of myocardial infarction [60]. CMVO can manifest as (i) early gadolinium enhancement (<2 minutes), with areas lacking gadolinium enhancement during the first passage of gadolinium through the cardiac tissue; or (ii) advanced gadolinium enhancement (after 10–15 minutes), in which the myocardial infarction area can be enhanced by gadolinium, while the area of microvascular obstruction cannot be strengthened and manifests as a dark area within the bright area. First-pass (early) CMVO is more sensitive than late CMVO because the latter does not sufficiently reflect the extent of CMVO [60, 61]. The presence of IMH is further indicated by T2*-weighted CMRI or T2* mapping [62]. As IMH occurs, erythrocytes extravasate into the myocardial tissue space, eventually leading to intraerythrocytic ferritin and hemosiderin, and iron degradation products can be detected using T2* imaging. Most studies use a cut-off value of T2* <20 ms to detect IMH [63, 64] (Fig. 2B). CMR seems to be a promising non-invasive imaging tool for assessing myocardial perfusion and flow quantification, boasting high spatial resolution, radiation-free operation, and high diagnostic accuracy. However, several limitations need to be highlighted, particularly the myocardial perfusion reserve index’s vulnerability to variations in resting perfusion and tissue contrast concentration.

#### 3.2.3 Positron Emission Tomography

Cardiac positron emission tomography (PET) stands out as the most reliable non-invasive diagnostic tool for identifying microvascular obstruction. By utilizing positron-emitting isotopes as tracers, PET boasts exceptional sensitivity and temporal resolution, enabling rapid dynamic visualization of tracer kinetics. The application of rest and stress PET facilitates the quantification of certain microvescular disease (MVD) indices, including myocardial blood flow (MBF), myocardial perfusion reserve (MPR, which represents MBF at peak stress), and myocardial flow reserve (MFR, calculated as the ratio of MBF during maximum coronary vasodilation to resting MBF). Notably, a myocardial flow reserve (MFR) of less than 1.5 is indicative of a diminished flow reserve, implying the presence of microvascular obstruction [65, 66]. Although PET is widely regarded as the gold standard for non-invasive microvascular function assessment, its adoption in clinical practice is restricted by certain limitations such as radiation exposure, limited availability and high costs.

### 3.3 Pathological Staining

Pathological staining of the heart is currently mainly used in large and small animal models. Thioflavin S staining is used as the gold standard for the diagnosis of CMVO, but owing to technical limitations associated with this staining method, it cannot be used clinically. Thus, the gold standard for the diagnosis of CMVO in clinical practice is still CMRI.

#### 3.3.1 Thioflavin S Staining

**Fig. 3.**
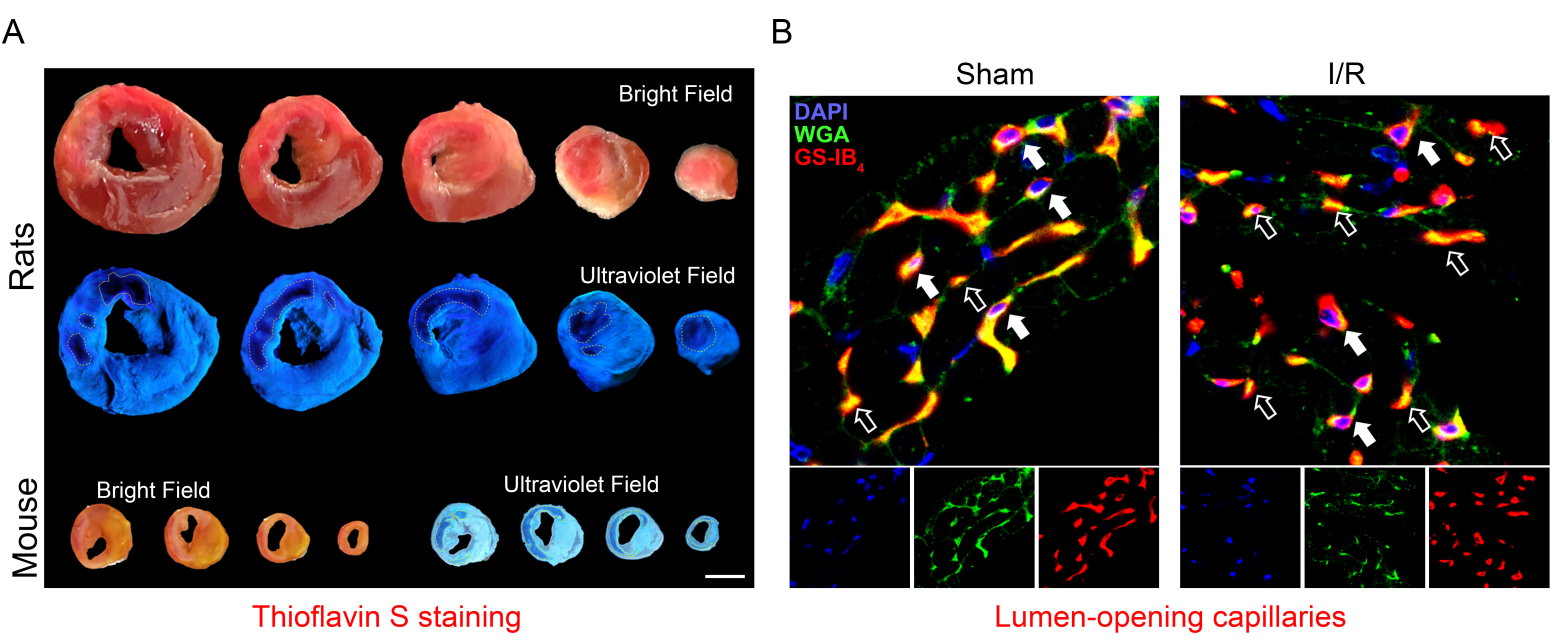
**Pathological staining of CMVO**. (A) The absence of thioflavin-S fluorescence, indicated by fine yellow lines, in representative cross-sectional images of the left ventricle (LV) indicates regions of no-reflow following I/R in rats (1 h/24 h) and mice (4 h/24 h). (B) Representative immunohistochemical images demonstrate capillaries in normal myocardium or within the border zone of the infarct area in mice that underwent sham-operation or I/R (4 h/24 h). Lumen-open capillaries are denoted by solid arrows, while lumen-close capillaries are denoted by open arrows. CMVO, coronary microvascular dysfunction and obstruction; DAPI, 4’,6-diamidino-2-phenylindole; WGA, wheat germ agglutinin; GS-IB_4_, griffonia simplicifolia-tetrameric type I isolectin B4; I/R, ischemia/reperfusion.

Thioflavin S staining is a method that involves the injection of sulfur S (4%) staining solution into the living heart. The stain after the aorta was clipped can be stained with the blood flow of the entire heart, and the no-reflow area due to no blood perfusion, the dye cannot enter, and then the heart was removed for sectioning, through the illumination of ultraviolet lamps, the area with sulfur can be colored, and the area without reflow manifests as a dark area [43, 67] (Fig. 3A).

#### 3.3.2 Prussian Blue Staining

Prussian blue staining can be used to diagnose IMH because IMH is accompanied by the leakage of erythrocytes into the tissue space. After lavage of the heart, the ferritin and hemosiderin of the erythrocytes remaining in the interstitial space will be stained with Prussian blue. Therefore, Prussian blue is also commonly used in animal experiments to diagnose IMH [68].

#### 3.3.3 Counting of Open Capillaries

Gao *et al*. [67] used the fluorescein isothiocyanate-labeled wheat germ agglutinin staining method to stain cardiac tissue sections from mice with CMVO to reflect the degree of cardiac capillary damage and counted the proportion of open capillaries under a fluorescent microscope. This method is an indirect indicator of the degree of damage to the microcirculation of cardiac tissue [67] (Fig. 3B).

## 4. Hazards of Coronary Microcirculatory Disorders

Vascular reopening technology is now widely popularized, and most patients with AMI can undergo treatment with vascular reopening. However, PCI and coronary artery bypass grafting only contribute to the recanalization of large blood vessels. At present, about one-third of patients still have a poor prognosis after PCI, and these patients still have poor postoperative blood perfusion; that is, obstructed perfusion of the microcirculation.

A pooled analysis of seven clinical trials (AIDA STEMI, APEX-AMI, CRISP AMI, LIPSIAbciximab, LIPSIA-N-ACC, LIPSIA-STEMI, and INFUSE-AMI) was performed previously [69, 70, 71, 72, 73, 74, 75]. Patients with STEMI who had undergone CMRI with late gadolinium enhancement in these seven randomized controlled trials (n = 1688) were identified, and patients with CMVO were followed up for at least 6 months (n = 960). The analysis showed that the median microvascular occlusion (percent left ventricular myocardial mass) was greater in patients with all-cause mortality (1.59% [interquartile range 0.00%–5.53%]) than in patients without all-cause mortality (0.46% [interquartile range 0.00%–2.48%]) (*p* = 0.04). Heart failure hospitalization was also more common in patients with all-cause mortality (1.67% [interquartile range 0.53%–3.47%]) than in those without (0.45% [interquartile range 0.00%–2.49%]) (*p* = 0.001). There was no statistically significant difference in the rate of reinfarction events between those with and without all-cause mortality (0.93% [interquartile range 0.00%–2.43%] vs. 0.46% [interquartile range 0.00%–2.55%], respectively) (*p* = 0.57) [76]. The multivariate analysis revealed that CMVO was a strong independent predictor of the composite incidence of all-cause mortality, heart failure hospitalization, all-cause mortality, or heart failure hospitalization. In the multivariate analysis adjusted for CMVO and infarct area, CMVO remained significantly associated with all-cause mortality, but not with heart failure hospitalization [76].

## 5. Strategies for Cardiovascular Protection

### 5.1 Intravenous or Intracoronary Injection of Drugs

#### 5.1.1 Sodium Nitroprusside

Sodium nitroprusside releases nitric oxide to relieve microcirculatory vasospasm by activating guanylate cyclase in vascular smooth muscle [77]. Two meta-analyses have demonstrated the effectiveness of sodium nitroprusside in primary PCI for infarct-related artery flow recovery and myocardial perfusion recovery in patients with STEMI. Sodium nitroprusside also improves left ventricular ejection fraction and reduces rehospitalization [78, 79].

#### 5.1.2 Adenosine

Adenosine is an endogenous purine nucleoside that is used to treat CMD. It has multi-target complex effects, including vasodilation, activation of adenosine triphosphate-sensitive potassium channels, inhibition of platelet aggregation, and leukocyte activation [80, 81]. A previous meta-analysis demonstrated that intraoperative use of adenosine in patients with STEMI reduces the incidence of TIMI blood flow less than grade 3 [82]. In addition, clinical studies and meta-analyses have shown that intraoperative intravenous injection or intraoperative coronary administration of nicorandil can prevent intraoperative reflow/slow flow and reperfusion arrhythmias in emergency PCI, as well as improving myocardial perfusion and clinical prognosis [83, 84].

#### 5.1.3 Calcium Channel Blockers

Calcium channel blockers (CCBs) (verapamil, diltiazem, nicardipine) also have some potential efficacy in the treatment of cardiac microvascular obstruction. They can act on the calcium ion channels of vascular smooth muscle to promote vascular smooth muscle relaxation and coronary vasodilation, which can alleviate cardiac microcirculation disorders caused by vasospasm to a certain extent. There are also clinical studies that show that intracoronary nicardipine was demonstrated to be a safe and highly effective pharmacological agent to reverse no-reflow during PCI [85, 86, 87]. However, data and studies on CCBs are still insufficient to show a significant beneficial effect on the phenomenon of cardiac microvascular obstruction.

#### 5.1.4 Glycoprotein IIB/IIIA Inhibitors

Glycoprotein IIB/IIIA inhibitors are potent antiplatelet agents that inhibit platelet aggregation and have shown benefits in the era prior to the routine use of dual antiplatelet therapy. To date, no studies have demonstrated convincing benefits of glycoprotein IIB/IIIA inhibitors beyond standard treatment. However, the On-TIME-2 study suggests that pre-hospital initiation of tirofiban infusion can lead to ST-segment resolution and improved clinical outcomes after primary percutaneous coronary intervention [88]. During a 3-year period, 1398 patients were randomized, 414 in phase 1 and 984 in phase 2. Major adverse cardiac events at 30 days were significantly reduced (5.8% vs. 8.6%, *p* = 0.043). There was a strong trend toward a decrease in mortality (2.2% vs. 4.1%, *p* = 0.051) in patients who were randomized to tirofiban pre-treatment, which was maintained during the 1-year follow-up (3.7% vs. 5.8%, *p* = 0.08). No clinically relevant difference in bleeding was observed [89].

### 5.2 Oral Drugs

#### 5.2.1 Antiplatelet Drugs

Aspirin combined with P2Y12 receptor antagonists is the cornerstone of STEMI treatment. Ticagrelor reversibly binds to P2Y12 receptors. It protects microcirculatory function and improves myocardial perfusion by promoting adenosine synthesis, reducing the rate of adenosine degradation. Compared with clopidogrel, ticagrelor is associated with a lower incidence of intraoperative slow flow/no reflow in emergency PCI [90]. In addition to lowering blood lipids, statins reduce endothelial cell damage and microvascular inflammation, and have a protective effect on the microcirculation [91]. The results of one meta-analysis suggest that treatment with an intensive statin loading dose before primary PCI significantly reduces the risk of no reflow or slow flow after surgery, reduces adverse events, and improves long-term outcomes [92].

#### 5.2.2 Metoprolol

Metoprolol, as β receptor blocker, is effective in inhibiting sympathetic overactivation in acute myocardial infarction (Table 1). However, in large animal experiments, early metoprolol administration during acute coronary occlusion increases myocardial salvage. The extent of myocardial salvage, measured as the difference between myocardium at risk and myocardial necrosis, was associated with regional and global LV motion improvement [93]. In the METOCARD-CNIC (Metoprolol Role in Cardiac Protection During Acute Myocardial Infarction) study, metoprolol was administered by a time-dependent action prior to primary percutaneous coronary intervention (pPCI), reducing the degree of infarction, preventing adverse left ventricular remodeling, preserving systolic function, and reducing the rate of rehospitalization in heart failure [36].

**Table 1.** **Main drugs and dosages for the treatment of coronary microvascular obstruction**.

Medication	Drug category	Dosage	Side effects
Sodium nitroprusside	Nitrovasodilator	Intracoronary: 60–100 µg bolus	Bradycardia, hypotension
Adenosine	Purinergic receptor agonist	Intravenous: 70 µg/kg/min infusion Intracoronary: 100–200 µg bolus	Bradycardia, hypotension
Tirofiban	Glycoprotein IIB/IIIA inhibitor	Initial dose: 25 mcg/kg intravenous injection (IV) bolus	Bleeding
		Maintenance dose: 0.15 mcg/kg/min IV infusion	
Verapamil	Calcium Channel Blocker	Intracoronary: 100–500 µg bolus	Bradycardia, transient heart block
Ticagrelor	P2Y12 receptor antagonist	Loading dose: 180 mg (2 × 90 mg tablets)	Bleeding
		Maintenance dose: 90 mg twice daily	
Metoprolol	β receptor blocker	Oral: 50–200 mg daily (extended-release)	Hypotension, bradycardia
		IV: 1.25–5 mg (acute setting)	

### 5.3 Non-Drug Treatment Methods

#### 5.3.1 Intracoronary Hypothermia

Generalized hypothermia has been used as a treatment to reduce systemic hypoblood flow and cerebral reperfusion injury after cardiac arrest [94, 95]. However, there are still huge challenges in clinical translational applications. As a further improvement, intracoronary hypothermia therapy has been proposed. In a recent study on intracoronary hypothermia, Pei *et al*. [96] used a large animal model of myocardial ischemia-reperfusion injury and found that intracoronary hypothermia can effectively alleviate the infarct area and microcirculatory obstruction of pigs and can improve the cardiac function of the myocardial ischemia-reperfusion injury model [96]. In another isolated beating porcine heart model of acute myocardial infarction, intracoronary hypothermia demonstrated protection of myocardial mitochondrial integrity and was effective in reducing myocardial injury [97]. All these indicate that intracoronary hypothermia has great application prospects in the treatment of myocardial infarction and cardiac microvascular obstruction.

#### 5.3.2 Coronary Sinus Occlusion

Pressure-controlled intermittent coronary sinus occlusion (PiCSO) can improve blood flow to non-perfused infarct-associated myocardial regions by blocking venous drainage of the remaining and uninvolved coronary arteries. This shunt reduces subendocardial ischemia. Potential benefits were demonstrated in dog models of LAD occlusion and concurrent coronary sinus occlusion [98]. In a comparative study, 45 patients with anterior STEMI within 12 hours of symptom onset underwent pPCI + PiCSO (started after reperfusion; n = 45) and compared to the propensity score matching control cohort (n = 80) of INFUSE-AMI. Cardiac magnetic resonance showed a lower infarct size on day 5 after PiCSO, which also indicates that PiCSO has great potential as an adjunct to pPCI in the treatment of myocardial ischemia-reperfusion [99].

#### 5.3.3 Other Non-Drug Treatment Methods

The standardized operation of interventional surgery can reduce the occurrence of CMVO, increase effective reperfusion of the myocardium, and ultimately save the myocardial tissue. Approaches include reducing the use of contrast agent, avoiding complex procedures, selecting appropriate stents, and appropriately using drug-coated balloons. Moreover, the importance of cardiac rehabilitation and health education cannot be ignored. For example, psychological counseling better equips patients to treat their condition correctly, maintain a positive attitude, quit smoking, consume less alcohol, consume a reasonable diet, lose weight, and maintain a healthy lifestyle, which can all help to improve their condition.

With the increasing maturity of nano-diagnostics and nano-therapy technologies, different types of nano-drug delivery systems have been designed and applied to the treatment of cardiovascular diseases [100, 101]. In the treatment of coronary microvascular obstruction, if an appropriate drug delivery system can be designed, it will greatly promote the diagnosis and treatment of coronary microvascular obstruction, which also has potential application value in clinical translation.

## 6. Conclusions

CMVO is a common event after first-time PCI and is associated with a poor clinical prognosis in patients with STEMI. CMVO is a complex phenomenon with multiple pathogenic mechanisms that often occur in combination. The diagnosis and differentiation methods are also relatively complex. Although CMVO has been studied for many years, no treatment has currently shown significant efficacy in reducing clinical adverse events. Given the multifactorial nature of the pathogenesis of CMVO, the combined use of pharmacological and non-pharmacological treatments may provide a new therapeutic strategy for improving the outcomes of patients with CMVO.
